# Experiential retailing in cultural spaces: A case study of multisensory design in batik boutiques

**DOI:** 10.12688/f1000research.163519.1

**Published:** 2025-05-20

**Authors:** Eri Naharani Ustazah, Purwanita Setijanti, Arina Hayati

**Affiliations:** 1 Department of Architecture, Sepuluh Nopember Institute of Technology, Surabaya, East Java Province, Indonesia

**Keywords:** Store atmosphere, multisensory retail environment, consumer experience, stimulus-organism-response, batik boutique.

## Abstract

**Background:**

This study examines the role of multisensory design in shaping the spatial atmosphere of batik boutiques and its impact on consumer experience and purchasing behavior. Unlike conventional retail spaces, batik boutiques offer culture-based products for a specialized consumer segment, where shopping involves a highly immersive sensory experience. By analyzing sight, sound, touch, smell, and taste, this research explores how sensory stimuli influence emotional engagement, consumer perception, and decision-making within experiential boutique environments.

**Methods:**

This research adopts a qualitative case study approach to analyze experiential retailing in batik boutiques as a brand differentiation strategy. Using an intersubjective paradigm, the study explores sociocultural consumer engagement through semi-structured interviews, direct observations, and photo-elicitation techniques. Data were collected over six months in a batik boutique in East Java, Indonesia, involving purposively selected participants to capture long-term consumer interactions with the store atmosphere and sensory elements influencing their shopping experiences.

**Result and Conclusions:**

The findings indicate that batik boutiques serve beyond commercial spaces, offering culturally rich, multisensory experiences that enhance consumer attachment, emotional connection, and brand loyalty. Sensory engagement through lighting, music, scent, and tactile interactions significantly impacts consumer perception and purchase behavior. Additionally, nostalgia, identity projection, and social interaction reinforce prolonged engagement, with retail spaces functioning as cultural hubs for heritage appreciation. This study highlights the importance of sensory-driven retail design, demonstrating how store atmosphere influences consumer emotions, cognitive responses, and purchasing intentions within experiential fashion retailing.

## 1. Introduction

Store atmosphere has been recognized for its ability to evoke cognitive and emotional responses in consumers, making it a crucial aspect of any retail environment ( Duong et al., 2022; Kotler, 1974). Over time, store atmosphere has evolved beyond merely serving as a service provider to becoming an experience creator, offering positive and memorable engagements for customers ( Biswas, 2019). As a result, store atmosphere has been increasingly acknowledged as an essential component of the retail environment and the overall shopping experience ( Puccinelli et al., 2009). Moreover, properly stimulating sensory modalities within the store atmosphere can significantly influence positive emotions and consumer attitudes. The sensory marketing literature extensively discusses the significant relationship between store atmosphere and consumer emotional, behavioral, and cognitive responses ( Biswas et al., 2019; Duong et al., 2022; Roggeveen et al., 2020). Store atmosphere functions as a configurative retail environment, incorporating elements such as lighting, color, music, and scent to stimulate consumer perception and emotions, ultimately influencing purchase behavior ( Levy, 2008). Several prior studies have examined sensory stimuli elements, including sight (seeing), sound (hearing), scent (smelling), touch (feeling), and taste (tasting) ( Biswas et al., 2019; Krishna, 2012). While store atmosphere has been extensively explored in relation to sensory modalities and consumer behavior ( Duong et al., 2022; Roggeveen et al., 2020), there remains a need for further investigation from the perspective of architectural elements and interior design to fully understand its impact on the retail experience.

Previous studies have explored the role of architectural elements, interior design, and supporting facilities in shopping centers, demonstrating how store atmosphere influences consumer behavior ( Kusumowidagdo et al., 2012). Store atmosphere is simultaneously shaped by perceptions of multiple sensory elements, including tactile and auditory aspects in addition to visual elements. Furthermore, Pallasmaa (2024) highlights that consumers develop an overall emotional impression of a space through a composite and subjective reflection of these sensory aspects, a notion also emphasized by Griffero (2016). In the context of large fashion retailers in developed countries, stores have successfully provided consumers with experiential shopping opportunities, particularly in flagship stores (e.g., Anderson et al., 2010; Moore & Doherty, 2007). In contrast, small boutiques often face limited resources and pressures to convert recreational visits into actual purchases, posing an ongoing challenge ( Clarke et al., 2012). Over time, fashion boutiques have found that merely selling products is no longer sufficient; instead, they must offer valuable in-store experiences to attract and retain their target market ( Alexander & Nobbs, 2020; Baker et al., 2020). This also presents a significant challenge for the retail sector, as it must address the declining number of customers visiting physical stores, as noted by Quartier et al. (2021). Moreover, competition within the fashion retail industry is becoming increasingly intensified in the digital era, with the rise of online stores and marketplaces further reshaping consumer shopping behaviors ( Bowstead, 2022).

To maintain a competitive advantage in the retail sector, particularly in fashion boutiques, retailers must strive to differentiate themselves through customer shopping experiences while emphasizing sensory elements within the store atmosphere as a key instrument. Consequently, there has been a growing demand for retail designers to create physical stores that evoke valuable customer experiences ( Dhruv et al., 2017; Servais et al., 2022). Findings from Clarke et al. (2012) indicate that not only tangible cues influence customer experiences, but also scent and lighting, which serve as defining characteristics of fashion boutiques. Supporting this, Quartier et al. (2014) reveal that store environment elements, particularly lighting and fragrance, significantly impact consumer perception, emotional experiences, and purchasing behavior. Meanwhile, Servais et al. (2022) argue that the combination of tangible aspects results in diverse consumer perceptions, representing a higher conceptual level of customer experience. However, no systematic approach has been established for physical retail design that fully integrates sensory elements to enhance fashion retail store visits ( Servais et al., 2022). Additionally, understanding consumer motivation can inform creative expressions in retail space design ( Klingmann, 2010). Consumer motivation can be classified as functional or hedonic, and in the context of fashion shopping, consumers tend to seek experiences beyond mere product acquisition to express their identity through fashion choices ( Alexander, 2019; Lashkova et al., 2020; Szocs et al., 2023). These studies collectively strengthen the examination of the impact of atmospheric space on consumers, offering a more comprehensive understanding of how physical environments can be optimized to influence customer perception and behavior.

The application of experiential sensory elements relevant to fashion boutiques plays a crucial role in shaping store atmosphere and architectural expression within retail spaces. In fashion retail, store environments are designed to increase the frequency and duration of customer visits, thereby enhancing the likelihood of purchases ( Clarke et al., 2012; Overdiek, 2018). Research suggests that customers who spend more time in-store are more likely to complete a transaction compared to those who stay for a shorter period ( Lindeman, 2007; Szocs et al., 2023). Fashion shopping can be categorized as hedonic or recreational, where consumers seek shopping experiences beyond mere product acquisition to establish self-image and identity ( Servais et al., 2022; Szocs et al., 2023). This reflects the consumer group’s taste identity, shaped by values and lifestyle perspectives. Understanding consumer motivation is thus essential, as it can inform creative expressions in retail space design ( Servais et al., 2022). In Indonesia, fashion boutiques cater primarily to the upper-middle-class to premium market segments, offering fashion products that incorporate ethnic cultural elements and exclusivity. Therefore, it is essential to conceptualize how consumer experiences within batik boutiques influence purchasing decisions. In the fashion boutique segment, customer experiences are typically multisensory, engaging all senses during the shopping process ( Clarke et al., 2012; Ustazah et al., 2024). Consumer experience encompasses not only tangible products but also intangible emotional values that customers perceive. Given the vital role of store atmosphere in shaping consumer behavior, emotions, interests, attitudes, and values, it is crucial to examine how spatial features are constructed and how they relate to the emotional, sensory, and behavioral objectives of commercial spaces ( Ustazah et al., 2024; Yoo et al., 1998). This study aims to determine how sensory consumer experiences in batik boutiques are a combination of multiple sensory inputs, forming patterns that support both the social and economic functions of commercial spaces.

Although numerous studies have identified the role of multiple sensory elements in shopping experiences (e.g., Duong et al., 2022; Roggeveen et al., 2020; Srinivasan & Srivastava, 2010), research exploring the potential of multisensory perception to enhance retail atmosphere in the context of fashion boutiques remains limited. Additionally, while existing studies have focused on large-scale fashion stores in developed countries (e.g., Biswas et al., 2019; Clarke et al., 2012), research on fashion retail environments in developing countries such as Indonesia remains scarce. These prior studies offer valuable insights into how atmospheric elements interact and are perceived by consumers, presenting a research gap that requires further exploration in the context of emerging markets. Furthermore, Nuralam et al. (2024) highlight that consumer characteristics and behaviors in emerging markets differ significantly from those in advanced markets, as they are more susceptible to information asymmetry. Therefore, it is essential for retailers to consider interior design as a strategic tool for developing a comprehensive understanding of consumer segmentation and for integrating sensory elements into the design process. Retail designers play a critical role in translating consumer needs into spatial programs ( Quartier, 2016). In the fashion shopping context, consumers tend to pursue motivations beyond product acquisition, seeking to express a particular self-image through their fashion choices ( DeNora & Belcher, 2000; Kaiser, 1997). This reflects consumer taste identity, shaped by their values, perspectives, and lifestyles. Understanding these dynamics is essential for designing immersive and culturally relevant retail environments, particularly in developing markets, where consumer engagement strategies must be adapted to local preferences and behaviors.

## 2. Literature review

### 2.1 Stimulus-Organism-Response framework

The Stimulus-Organism-Response (S-O-R) framework, initially proposed by Mehrabian and Russell (1974), provides a valuable theoretical lens to examine how store atmosphere influences consumer behavior. This model conceptualizes consumer response as a result of environmental stimuli (S) influencing internal states (O), which in turn elicit behavioral reactions (R). In retail settings, especially fashion boutiques, the stimulus refers to multisensory atmospheric elements such as visual design, scent, music, and layout that create the store environment ( Lazaris et al., 2022; Mower et al., 2012). These cues are purposefully curated to provoke emotional and cognitive responses that can positively influence consumer behavior, including purchase intentions, store loyalty, and engagement ( Huynh et al., 2024; Vieira, 2013). The relevance of this framework has been validated by several contemporary studies in retail marketing, including those by Lazaris et al. (2022) and Vieira (2013), which demonstrate that affective responses like pleasure and arousal mediate the relationship between sensory stimuli and consumer behaviors. Fashion boutiques, by their very nature, serve as platforms for hedonically motivated shopping, where identity, emotional fulfillment, and lifestyle alignment become paramount. The S-O-R model, therefore, enables a systematic understanding of how architectural elements and sensory cues coalesce to create an engaging in-store experience.

Emotions such as pleasure and arousal play a mediating role between sensory cues and consumer actions. The organism component in the S-O-R model encapsulates these emotional and cognitive responses that are activated by the store’s atmosphere. For instance, lighting and color schemes can influence mood and perceived quality, while scent and music enhance the immersive character of the shopping experience ( Roschk et al., 2017; Spangenberg et al., 2005). The response phase typically manifests in longer store visits, increased impulse purchases, and stronger brand attachment ( Donovan et al., 1994; Helmefalk & Hultén, 2017). Berman and Evans (2004) outline the core components of a retail environment—exterior, general interior, layout, and displays—that serve as crucial stimuli in shaping the customer journey. The exterior includes signage and window displays that generate the first impression, while the general interior (lighting, color, music, scent) shapes the emotional ambiance. The store layout, including furniture arrangement and traffic flow, contributes to ease of navigation and product accessibility. Displays and thematic decorations serve to engage consumers visually and encourage exploration. These elements align with the S-O-R structure, where thoughtful spatial planning acts as stimuli that influence affective and cognitive organismic states, leading to behavioral responses.

In the context of fashion boutiques, the S-O-R framework facilitates an in-depth understanding of how consumer motivation is translated into spatial design and multisensory experiences. Boutique stores operate under unique spatial and economic constraints compared to large-scale retailers but rely heavily on atmospheric strategies to differentiate themselves and communicate brand identity ( Alexander & Nobbs, 2020; Clarke et al., 2012). The application of S-O-R enables researchers and designers to decode how these constrained environments can still generate high-impact consumer experiences. For example, aroma congruent with visual cues, ambient lighting that complements store themes, and curated music can all enhance emotional engagement, leading to elevated purchase intent ( Helmefalk & Hultén, 2017; Lazaris et al., 2022). This is particularly pertinent in developing markets like Indonesia, where emerging consumer segments are increasingly responsive to holistic and culturally attuned shopping experiences ( Nuralam et al., 2024). Moreover, the S-O-R model acknowledges that emotional and cognitive responses to atmospheric stimuli are shaped by cultural, social, and psychological motivations. These include aspirations for identity expression, social signaling, and affective resonance with brand aesthetics ( Khan et al., 2024; Prentice & Loureiro, 2018). Thus, S-O-R is not only a descriptive framework but also offers prescriptive insights for enhancing retail design in alignment with consumer expectations. By leveraging this theoretical lens, the study provides a comprehensive understanding of the mechanisms through which fashion boutique atmospheres influence shopper behavior and contribute to experiential retail value.

### 2.2 Psychological process on physical environment in influencing consumer experience

Retail design has emerged as a relatively new discipline within academic research over the past two decades ( Quartier et al., 2014; Petermans & Kent, 2017), yet it remains underexplored in scholarly literature. Specifically, the relationship between store atmosphere and psychological processes in shaping consumer experiences has received limited attention, despite its critical role in physical environment design ( Servais et al., 2022). Nevertheless, several studies have considered marketing sources in the omnichannel approach, which presents opportunities for integration within retail design (e.g., Servais et al., 2018, 2022). Furthermore, the interconnection between marketing sources and retail design is carefully evaluated to focus on different objectives, methods, and terminologies in creating consumer experiences ( Servais et al., 2018). These experiences are examined from the perspective of sensory systems, where store atmosphere functions as a stimulus receptor. Although Servais et al. (2022) highlight that retail design remains an underdeveloped area in academic literature, primarily because it is a practice-based applied field, only a limited number of studies have explored the relationship between store atmosphere and experiential sensory engagement (e.g., Clarke et al., 2012; Servais et al., 2022; Ustazah et al., 2024). Nevertheless, these insights serve as valuable contributions that warrant further examination in the study of store atmosphere and sensory experiences, particularly in understanding how environmental stimuli influence consumer behavior and perception.

In the field of store atmosphere and retail design, psychological processes in marketing are often associated with customer reactions to the physical retail environment, emphasizing emotional rather than cognitive perception, particularly in the context of hedonic consumption ( Clarke et al., 2012; Petermans & Kent, 2016; Quartier et al., 2014). This is closely related to the extent to which sensory stimulation in the store atmosphere contributes to emotional expression and emotional exchange, which are shaped through customer interactions. As highlighted in Pal et al. (2025), shopping experiences are formed through a complex interaction of social, physical, and psychological factors, which collectively shape customers’ collective perception during their visit. In this context, psychological processes are explained through affective and cognitive responses to sensory experiences ( Spence et al., 2014), which in turn influence consumer decision-making processes related to store engagement and purchasing behavior ( Lashkova et al., 2020).

Referring to the model developed by Mehrabian and Russell (1974), emotions triggered by a particular environment can be classified into three dimensions: pleasure, arousal, and dominance, which describe individuals’ feelings and their approach or avoidance behaviors toward a given space. The Stimulus-Organism-Response (S-O-R) paradigm provides a practical conceptual framework for linking environmental factors (Stimulus) with individual behavioral responses (Response) through internal processes (perception, cognition, and emotional mechanisms) of an individual (Organism). In the context of store atmosphere, retailers use sensory stimuli to create a pleasant store ambiance, ultimately increasing purchase intentions, which are part of consumer behavior ( Helmefalk & Hultén, 2017; Lashkova et al., 2020; Spence et al., 2014). Responses to environmental stimuli generally fall into two basic behavioral categories: approach and avoidance. Approach behavior (or convergence) occurs when individuals react positively to the environment, while avoidance behavior reflects negative reactions. The emotional state induced by the store atmosphere—pleasure, arousal, and dominance—is strongly associated with consumers’ desire to stay longer, shop, and engage in retail consumption ( Mehrabian & Russell, 1974).

### 2.3 Experiential fashion retailing: The multisensory dimensions of store atmosphere

The concept of store atmosphere has long been a crucial component in marketing research, particularly in understanding consumer behavior and preference formation ( Darden & Babin, 1994; Johnstone, 2012). However, the exploration of consumer interaction within “servicescapes”—a term often used interchangeably with “retail environment”—remains relatively underdeveloped ( Alexander, 2019; Johnstone, 2012). A retail environment is defined as a space where goods and services are directly sold to consumers ( Johnstone & Todd, 2012; Kotler, 1973). Traditional marketing research predominantly focuses on stimulus-response interactions within retail settings, yet recent developments highlight the significance of multi-sensory experiences, engaging all five human senses—sight, sound, smell, taste, and touch ( Krishna, 2012). A multi-sensory experience is the outcome of sensory reactions to various marketing stimuli. Visual components, auditory cues, and flavors contribute significantly to shaping brand identity and reinforcing its image in consumers’ minds ( Krishna et al., 2010). Among these, scent plays a pivotal role in evoking emotional responses and solidifying long-term brand associations ( Lashkova et al., 2020). Additionally, tactile engagement enhances customer-product interaction, increasing the likelihood of impulsive purchases ( Lashkova et al., 2020). The integration of all five senses fosters a holistic multisensory experience, enriching the overall perception of a retail establishment ( Spence et al., 2014).

Research supports the impact of various sensory elements on consumer perception and behavior. Studies on visual appeal have examined the influence of colors ( Puccinelli et al., 2013; Baek et al., 2018), lighting effects ( Clarke et al., 2012; Lashkova et al., 2020), and visual complexity ( Jang et al., 2018) in shaping store atmosphere. Scent is another crucial factor; even a fleeting exposure to an aroma can trigger sensory activation and emotional response, enhancing the consumer experience ( Clarke et al., 2012). Additionally, auditory stimuli, such as music, significantly influence consumer engagement and strengthen brand identity ( Servais et al., 2022; Szocs et al., 2023). In contemporary fashion retailing, an experiential approach to store atmosphere is imperative for establishing a compelling brand presence. By strategically integrating multisensory elements, retailers can create immersive environments that not only attract but also retain customers. This approach moves beyond traditional marketing paradigms, fostering deeper emotional connections between consumers and brands. Ultimately, experiential fashion retailing underscores the importance of sensory engagement in driving consumer behavior, enhancing brand loyalty, and differentiating retail spaces in an increasingly competitive market.

Sight - Sight plays a fundamental role in shaping consumer perception within retail environments. Visual elements such as color, lighting, and store layout significantly influence consumer emotions and decision-making processes. Studies indicate that warm colors like red and orange can evoke excitement and urgency, while cooler tones like blue and green promote a sense of relaxation ( Puccinelli et al., 2013). Additionally, appropriate lighting enhances product visibility and creates an inviting ambiance, impacting consumers’ time spent in the store ( Clarke et al., 2012; Lashkova et al., 2020). Visual complexity, such as the arrangement of merchandise and the overall store design, also affects how customers engage with a retail space, reinforcing brand identity and aesthetic appeal.

Sound - Sound serves as an essential sensory component that affects the overall shopping experience. Background music, tempo, and volume can significantly influence consumer emotions and purchasing behavior. Research indicates that slow-tempo music encourages customers to spend more time browsing, whereas fast-tempo music can create a sense of urgency and excitement ( Biswas, 2019). Additionally, music genre alignment with brand identity helps reinforce a retailer’s image and appeal to a specific target market ( Biswas, 2019; Biswas et al., 2019). The strategic use of soundscapes, such as nature-inspired audio or curated playlists, further enhances store ambiance, fostering positive associations and improving customer satisfaction.

Smell - Scent has a profound effect on consumer perception and brand recall. Studies suggest that pleasant aromas can elevate mood, increase time spent in stores, and enhance product evaluations ( Clarke et al., 2012). Certain scents evoke specific emotions and memories, making them powerful tools for establishing long-term brand connections. For instance, floral scents are often associated with femininity and elegance, while woody or musky fragrances create a sense of warmth and sophistication ( Biswas et al., 2019; Krishna, 2012). By incorporating signature scents in retail spaces, brands can create immersive experiences that differentiate them from competitors and reinforce brand identity in consumers’ minds.

Taste - Taste is an often-overlooked sensory element in fashion retail but plays a role in enhancing consumer engagement, particularly in luxury or experiential retail environments. Complimentary beverages or gourmet samples provide a sense of exclusivity and hospitality, encouraging customers to spend more time in stores. Studies suggest that taste can evoke emotions and create a multisensory memory, associating positive flavors with a brand’s identity ( Krishna, 2012; Krishna et al., 2010). Retailers incorporating in-store cafes or offering refreshments enhance customer satisfaction, making the shopping experience more enjoyable and immersive. This strategy is particularly effective in high-end retail spaces where customer engagement and brand perception are paramount.

Touch - Touch is a crucial sensory factor that influences purchasing behavior by fostering a sense of connection between the consumer and the product. The ability to physically interact with merchandise increases purchase intention and encourages unplanned buying decisions ( Biswas et al., 2019; Krishna, 2012). Textures, fabric quality, and product weight contribute to consumers’ evaluations, making tactile engagement essential in fashion retail. Providing opportunities for hands-on interaction through display setups, fitting rooms, and product trials enhances customer experience. Furthermore, the psychology of touch extends beyond products to store furnishings and packaging, reinforcing brand identity through high-quality materials and ergonomic designs that elevate the perceived value of a brand.

## 3. Method

### 3.1 Research design

This study employs a qualitative case study approach to explore the application of experiential retailing in fashion boutiques as a strategy for brand differentiation. Given the limited qualitative investigations into how fashion retailers implement experiential strategies, this study adopted a case study approach to enable an in-depth, context-rich exploration of the phenomenon. Specifically, the research involved purposively selected fashion boutiques that specialize in batik, chosen due to their multisensory retail settings and cultural positioning. This approach allowed for the collection of detailed data from multiple sources—interviews, observations, and photo-elicitation—enabling triangulation and a deeper understanding of how spatial, sensory, and symbolic elements are employed to create unique brand experiences. The use of photo-elicitation enhances data representation by incorporating visual media, allowing participants to articulate their sensory experiences more effectively ( Biehl-Missal, 2013). This method is particularly useful in capturing the aesthetic, emotional, and cognitive responses to boutique spatial design, contributing to a holistic analysis of experiential fashion retailing. Data collection was conducted over a six months period using a consistent protocol across all cases to control for variability, and attention was given to potential researcher bias through reflexive journaling and member checking. The research focuses on batik boutiques, which provide a multisensory shopping experience where consumers engage with cultural products in a unique retail environment. The study adopts an intersubjective paradigm, emphasizing the sociocultural engagement of consumers within the boutique setting ( Groat & Wang, 2013). This paradigm frames knowledge through an understanding of how individuals interact with and experience the retail environment. The research ensures reliability and validity through careful participant selection, data consistency, and rigorous interpretation of findings.

### 3.2 Data collection and participants

Data collection was conducted over six months through qualitative methods, including semi-structured and in-depth interviews, direct observation, and photo-elicitation techniques ( Creswell & Clark, 2017; Yin, 2017). Participants were purposively selected based on recommendations from the boutique owner to ensure a representative sample of long-term customers. The interviews were designed with open-ended questions to encourage detailed responses. Observational data were systematically documented in field journals, capturing spatial interactions and consumer behavior. This study examines a batik boutique in East Java, Indonesia, which serves as a case study for understanding experiential retailing in the fashion sector. The boutique, established in 2004, specializes in high-quality batik products with exclusive designs that blend classical and contemporary motifs, particularly those inspired by Surabaya City. Catering to an upper-middle-class clientele, including professionals, dignitaries, and art collectors, the boutique offers more than just retail space; it functions as a cultural and educational hub where customers can engage in workshops and learn about batik craftsmanship.

Methodological triangulation was employed to enhance reliability by cross-referencing data from interviews and observations. Data transcription was facilitated using professional software, and participants were given opportunities to review their transcripts to ensure accuracy. Photo-elicitation was used as a key technique, where participants selected three preferred boutique spots that influenced their purchasing decisions. This method allowed for deeper exploration of sensory perceptions, moods, and spatial experiences ( Biehl-Missal, 2013). Ethical considerations were prioritized, with verbal and written consent obtained from participants. This study received ethical approval from the Directorate of Research and Community Service (Direktorat Riset dan Pengabdian kepada Masyarakat/DRPM), Institut Teknologi Sepuluh Nopember, Surabaya, Indonesia, under Approval No. 1969/IT2.IV.1/B/TU.00.09/2023, dated 22 May 2023. The research strictly adhered to the ethical principles outlined in the Declaration of Helsinki ( World Medical Association, 2013), which governs ethical conduct in research involving human subjects ( https://www.wma.net/policies-post/wma-declaration-of-helsinki-ethical-principles-for-medical-research-involving-human-subjects/). Prior to participation, all individuals were thoroughly informed about the study’s purpose, procedures, potential risks, and their rights as participants. Written informed consent was obtained from each participant, confirming their voluntary participation and understanding of the study. Participants were assured that their identities would remain confidential and anonymous. All data were securely stored on password-protected devices accessible only to the research team and used solely for academic and scientific purposes within the scope of this study.

Participants remained anonymous, and all identifiable information was coded. Five participants were selected, all of whom had been loyal customers for over a decade. Their backgrounds varied, including socialites, art enthusiasts, government officials, and banking executives, representing the boutique’s target market (see Table 1). The analysis identified consistent patterns in sensory perception mechanisms, with follow-up interviews conducted to validate findings and ensure data reliability. To find out the customer’s experience, the following Table 2 presents the structure of the questions used in the in-depth interviews, which refers to the structure of the questions in the multisensory perception theory of fashion retail ( Alexander & Nobbs, 2016).

**Table 1.** List of Participants.

Code	Description	Gender & Age	Customer Segment	Loyalty Duration
P1	A socialite	She/50	Luxury and high-end customers	Over 13 years
P2	A fine arts lecturer	She/50	Cultured and intellectual customers	Over 12 years
P3	A socialite and bank director	She/50	Professional and affluent customers	Over 10 years
P4	Head of a state government office	She/52	Conservative and professional customers	Over 12 years
P5	A socialite and medical beauty doctor	She/45	Health and beauty-conscious customers	Over 10 years

**Table 2.** Structure of questions for in-dept interviews with Batik Boutique participants.

Aspects	Description
Mood initial statement	What is your mood like before you enter the store?
Systematic exploration of spatial elements that stimulate each of the following sensory expression: sight/sound/smell/ touch/taste	What are the elements in the selected area that trigger a sight/sound/smell/touch/feel experience that leaves an impression?
Systematic exploration of each sense sensations: Sight/ sound/smell/ touch/taste	What emotional response is elicited from sight/ sound/smell/ touch/taste sensors?
A systematic exploration of retail space behavior	What activities and behaviors are performed in the selected space area of the space that leave a lasting impression?
Mood status when exiting the room	What is your mood when you walk out of the store?

### 3.3 Data analysis

The data analysis process was conducted using NVivo 12, a proprietary computer-assisted qualitative data analysis software developed by QSR International ( https://www.qsrinternational.com/nvivo-qualitative-data-analysis-software/home), to ensure systematic coding and thematic interpretation of qualitative data. NVivo facilitated the organization, coding, and retrieval of rich textual and visual data collected from interviews, observations, and photo-elicitation. To ensure accessibility and transparency in future replications, researchers may alternatively use RQDA, an open-source R-based qualitative data analysis tool ( Zha et al., 2022), which provides comparable functions for coding, memoing, and query analysis (available at https://rqda.r-forge.r-project.org/). The primary coding framework was developed based on key themes, including the boutique’s physical elements, sensory perceptions, emotional responses, and spatial behavior. The process involved segmenting data into specific nodes, allowing for structured thematic analysis ( Miles et al., 2019). A matrix coding query was utilized to examine relationships between various aspects of the retail environment and participants’ sensory experiences. This approach enabled the identification of associative patterns linking boutique elements to consumer emotions and behaviors. The data visualization and coding process provided saturation, ensuring reliability by demonstrating consistent themes across multiple participants (see Table 3).

**Table 3.** Dimensions of Retail Atmosphere Elements Influencing Sensory Experience.

	(P1)	(P2)	(P3)	(P4)	(P5)
Store Layout and Design	33,15%	38,14%	33,24%	38,71%	44,97%
Display and Decoration	31,75%	30,57%	38,34%	29,62%	38,79%
General Interior	21,94%	25,04%	21,18%	30,62%	13,22%
Exterior	13,16%	6,26%	7,24%	1,06%	3,02%

The results indicated a hierarchical influence of boutique elements on sensory experience. Layout and design emerged as the most dominant factor, with percentages ranging from 33.15% to 44.97% across participants. Display and decoration also had a strong impact, ranking second in significance, with a percentage range of 29.62% to 38.79%. General interior aspects displayed moderate influence, varying between 13.22% and 30.62%. Exterior elements had the least impact, ranging from 1.06% to 13.16%, indicating their minimal role in shaping sensory perceptions. Despite this, the analysis confirmed data saturation, as patterns remained consistent across participants. NVivo facilitated cross-referencing of interview data, enhancing accuracy and depth in identifying critical factors affecting the boutique’s experiential atmosphere. The structured presentation of results allowed for a comprehensive understanding of the sensory dynamics within the retail space.

## 4. Result and analysis

The objective of this research is to explore and enhance experience-based multisensory perception. The discussion of multisensory perception in spatial atmosphere focuses on understanding perception through different senses, determining which senses are engaged first, identifying dominant sensory modalities, analyzing the influential and most perceptible elements, assessing the composition of these elements, and examining their relationships at the cognitive stage ( Servais et al., 2022). Furthermore, the discussion on the process mechanism model is based on a conceptual framework that links environmental aspects (Stimulus) to individual behavioral responses (Response), mediated by internal processes such as perception, cognition, and emotion ( Servais et al., 2022). To obtain comprehensive data and research findings, three selected areas were identified in the research procedure. These areas, which are depicted in the plan below, serve as the basis for analyzing the spatial experience within the boutique environment.

**Figure 1.**
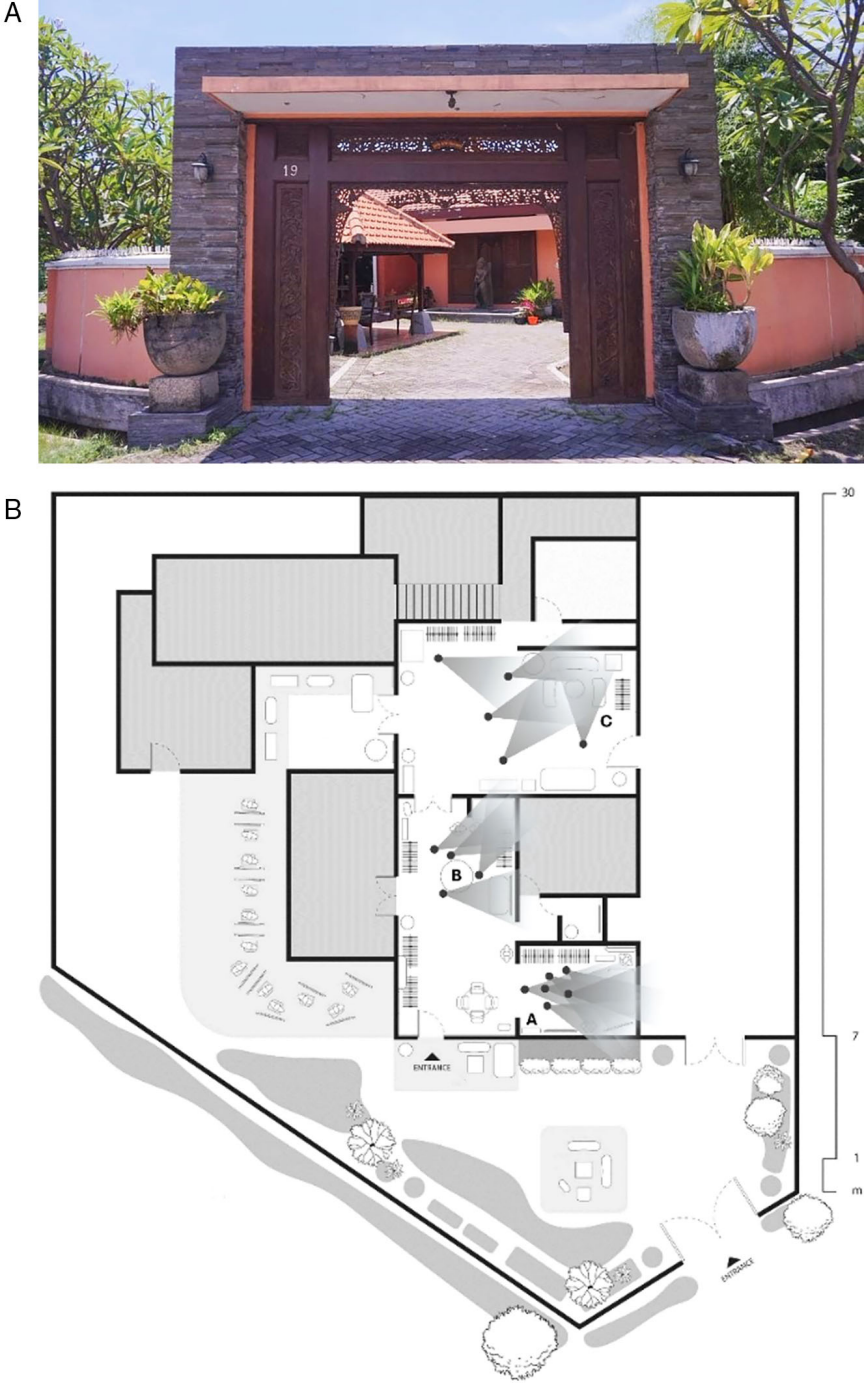
The front of the boutique and point plan of participants' selected areas of research study locations. Note: Figure 1 is an original creation by the authors, specifically designed for this study.

The collection of angles chosen by the participants is used to identify areas where elements relevant to the research discussion are present. The following are the specific corners of the room where participants took photos. Three primary areas were identified, designated as Areas A, B, and C, and their positions were mapped within the perception process and stimulus elements of the room plan (see Figure 1). The sensory perception experienced by participants within the batik boutique space was explored through in-depth interviews on sensory experiences, incorporating three selected photographs that captured their preferred angles, depicting the spatial design and atmosphere of the retail space. These perceptions are then analyzed in a sequence that explains the stimulus elements observed and noted by the participants. The following section presents the results of data collected from participants, which were subsequently processed using NVivo. The process of identifying and classifying coded data was based on theoretical frameworks and the findings from in-depth interviews using the photo-elicitation technique (see Table 4).

**Table 4.** Operational Definitions in the Data Coding Process.

Spatial Atmospheres Aspect in the S-O-R Framework	Retail Atmospheres Dimension Based on Theory	Code of Spatial Atmospheres Element Based on Participant Experiences Data	
Retail Environmental stimuli (Stimulus)	Exterior	1. Entrance 2. Window/opening 3. Building height 4. Garden	
	General Interior	1. Brightly painted walls 2. Terrazzo floor 3. Color schema 4. Room temperature 5. Natural and artificial lighting	
	Store layout and design	1. Additional carpet in the product area 2. Teak wood chairs situated around a teak wood table 3. Configuration of a teak wood sofa and table 4. *Gebyok* (ornate wooden panel) 5. Mirror with a teak wood stand 6. Teak wood credenza	
	Display and Decoration	1. A *mben* (traditional teak wood bench as display platform) 2. *Gawangan* (carved teak wood as display stand) 3. *Gebyok* (ornate wooden panel) 4. Teak wood credenza for displaying photo frames 5. Batik fabric decoration 6. *Artwork* (decorative plates, ceramics) 7. Large teak wood *frame* carved mirror on the wall 8. Painting	
Sensory Perception (Organism)	Sight	Seeing, looking, observing, selecting, photographing	
	Sound	Listening, sounds of movement, sounds of friction	
	Touch	Holding, wearing, stroking, sticking, wearing on the body, reaching, exposing, uncovering, selecting	
	Smell	Smelling, sniffing, savoring scents	
	Taste	Sipping a drink	
Emotional Status (Organism)	Pleasure	1. Fun 2. Comfortable 3. Mesmerized 4. Perceived *adem* (comfortable, satisfied) 5. Perceived natural (fresh, satisfied) 6. Perceived antique (uniqueness, beauty) 7. Warm	
	Arousal	1. Familiar 2. Relaxed 3. Perceived homey (familiarity, togetherness) 4. Perceived artistic (admiration, inspiration) 5. Passion 6. Perceived traditional (connectedness, pride, nostalgia) 7. Perceived cultured (connectedness, pride, respect)	
	Dominance	1. Feel free 2. Familiar (confident and interactive) 3. Perceived homey (free and interactive)	
Consumer Behavior (Response)	Approach response	1. Linger/stay to enjoy the product and ambience setting 2. Interact and engage with the product by observing, feeling, and wearing it according to traditional procedures with the product 3. Allow time to enjoy and imagine the atmosphere of the house 4. Move around freely between different locations within the establishment 5. Sitting comfortably and freely 6. Interacting and engage in social interaction with colleagues and clients. 7. Reminisce and engage with the interior design elements 8. Make unplanned purchases 9. Taking pictures and capturing moments 10. Reflecting on the mix and match and showing off with the product 11. Relax and enjoy refreshments	
	Avoidance response	No avoidance response	

Subsequently, the coded data is presented in the data coverage analysis. The data coverage facilitates a comparison of coding distribution across various categories, including the retail atmosphere dimension, sensory perception, emotional response, and consumer behavior. The following section presents the results of the data coverage analysis, focusing on the spatial atmosphere in the three selected areas (see Table 5). Furthermore, this study consolidates data to generate themes that represent the intended data, ensuring their applicability. The research draws upon the Theory of Consumer Behavior in the retail sector ( Rangkuti, 2011; Pine & Gilmore, 1998) to conduct an in-depth exploration based on the identified themes. The findings reveal relationships and similarities in interpretation, function, and form, which are consolidated to unify and strengthen the meaning and connections between two or more data points, thereby forming a more robust entity (see Table 5). Consequently, themes derived from the Theory of Consumer Behavior emerge and are analyzed (see Table 6).

**Table 5.** Consolidation of Consumer Behavior Themes.

Code of Behavioral Response Patterns	Consolidation of Behavioral Responses	Behavioral Response Theme
Interacting and engaging with the product by observing, feeling, and wearing it according to traditional procedures	Product interaction and social interaction ( Rangkuti, 2011)	Personal and social interaction
Interacting and engaging in social interaction with colleagues and clients		
Lingering/staying to enjoy the product and ambiance	Behavior encompasses not only the consumption of products but also all interactions, including the store atmosphere and service ( Pine & Gilmore, 1998)	Cultivating intention (developing motivation to support and enhance shopping desires and goals)
Relaxing and enjoying refreshments		
Moving freely between different locations within the establishment	Consumer behavior in a retail setting ( Pine & Gilmore, 1998); Interaction with the store ( Rangkuti, 2011)	Interaction with interior spaces and elements
Sitting comfortably and freely		
Taking pictures and capturing moments	Consumer engagement and personalization ( Pine & Gilmore, 1998)	Representation of identity and social affiliation
Reflecting on mix-and-match styles and showcasing the product		
Allowing time to enjoy and imagine the atmosphere of the boutique	Behavior influencing memory and immersion ( Pine & Gilmore, 1998)	Immersive and nostalgic experience
Reminiscing and engaging with the interior design elements		

**Table 6.** Results of Code Coverage of Batik Boutique Retail Atmosphere Dimension Elements Considered in the Participant's Experience (amount in percentage).

Aspects		Area A	Area B	Area C
Retail Atmospheres Dimension	Display and Decoration	33,87%	34,92%	25,10%
	Exterior	4,44%	5,25%	0,00%
	General Interior	27,82%	23%	27,57%
	Store Layout and Design	33,87%	36,44%	47,33%
Sense	Sight	53,24%	53,94%	43,85%
	Sound	24,29%	25,87%	30,05%
	Touch	16,80%	11,05%	12,30%
	Smell	5,67%	9,14%	7,49%
	Taste	0%	0%	6,31%
Emotional Dimension	Pleasure	68,21%	39,70%	41,57%
	Arousal	22,54%	28,14%	24,10%
	Dominance	9,25%	32,16%	34,34%
Behavioral response theme	Immersive and nostalgia	1,70%	20,75%	6,91%
	Personal and social interaction	34,59%	18%	30,55%
	Interaction with interior spaces and elements	10,21%	5,19%	8%
	Cultivating intention, (develop motivation to support and enhance shopping desires and goals)	17,77%	21%	29,82%
	Representation of identity and social affiliation	35,73%	35,30%	24,36%

The analysis of the retail atmosphere dimensions highlights key spatial elements influencing consumer experiences in the boutique batik setting. The display and decoration category received the highest focus in Area B (34.92%), closely followed by Area A (33.87%), with Area C lagging behind (25.10%). This suggests that Areas A and B emphasize aesthetic appeal and decorative elements, enhancing their attractiveness. In contrast, the general interior dimension exhibited similar levels of coding in Areas A (27.82%) and C (27.57%), while Area B had slightly less focus (23%). Meanwhile, store layout and design were most dominant in Area C (47.33%), followed by Area B (36.44%) and Area A (33.87%). The exterior dimension had minimal coding coverage across all areas, with Area C showing no engagement (0.00%) and Areas A and B demonstrating very low engagement (4.44% and 5.25%, respectively). This pattern indicates that spatial organization plays a crucial role in shaping the boutique experience, with an internal focus on ambiance rather than external appearances, ensuring that customers remain engaged once they enter the store.

The results of sensory perception analysis confirm that all five senses are engaged in participants’ interactions with the boutique environment. Sight emerged as the most dominant sense, with the highest coverage in Area B (53.94%), followed closely by Area A (53.24%) and Area C (43.85%). This indicates that visual stimuli play a crucial role in participants’ experiences, particularly in Areas A and B. Sound perception was most pronounced in Area C (30.05%), followed by Area B (25.87%) and Area A (24.29%), suggesting that distinctive auditory elements are more noticeable in Area C. The sense of touch had the highest engagement in Area A (16.80%), with lower focus in Area C (12.30%) and Area B (11.05%), possibly due to material and design elements present in Area A. Smell perception was relatively low across all areas, with Area B leading (9.14%), followed by Area C (7.49%) and Area A (5.67%), indicating that scent played a minor but slightly more pronounced role in Area B. Notably, taste was only recorded in Area C (6.31%), making it a unique feature that was absent in Areas A and B. The differences in sensory perception across areas suggest a tailored approach to spatial experience, enhancing the engagement of visitors through varying sensory stimuli.

The emotional response analysis further reveals how spatial design influences participants’ feelings. Area A registered the highest level of pleasure (68.21%), far exceeding Area B (39.70%) and Area C (41.57%), indicating a focus on comfort and aesthetics. Area B exhibited the highest level of arousal (28.14%), suggesting a more stimulating environment compared to Area C (24.10%) and Area A (22.54%). Meanwhile, dominance was strongest in Area C (34.34%), followed closely by Area B (32.16%), with Area A showing the lowest engagement (9.25%). These variations in emotional response align with specific spatial and design strategies, with Area A offering a relaxing and pleasurable experience, while Areas B and C evoke engagement and a sense of control. The emphasis on different emotional triggers across the three areas demonstrates a deliberate approach to crafting a multi-faceted boutique experience, ensuring that the space appeals to a diverse range of consumer preferences.

The consumer behavioral response themes illustrate how participants interact with the boutique environment. Personal and social interaction emerged as a dominant response in Area A (34.59%) and Area C (30.55%), while Area B had a lower engagement (18%). Representation of identity and social affiliation was significant across all areas, particularly in Area A (35.73%) and Area B (35.30%), with a slightly lower engagement in Area C (24.36%). The theme of immersive and nostalgia had the highest focus in Area B (20.75%), with much lower emphasis in Areas C (6.91%) and A (1.70%), indicating that Area B fosters an environment that evokes strong emotional and nostalgic connections. Interaction with interior spaces and elements showed moderate engagement, with the highest response in Area A (10.21%), followed by Area C (8.00%) and Area B (5.19%). The theme of cultivating intention, which relates to developing motivation to support and enhance shopping desires and goals, was most pronounced in Area C (29.82%), followed by Area B (21.00%) and Area A (17.77%). These findings suggest that each area within the boutique is designed to elicit distinct behavioral responses, reinforcing the strategic spatial organization aimed at enhancing consumer engagement and brand experience.

Subsequently, the interconnections between code categories (nodes) were examined using a query matrix (see Table 7). This investigation aimed to identify data patterns, explore the emergence of themes or topics, and elucidate relationships between various aspects. In this study, a query matrix was constructed to analyze the relationships between elements of the retail atmosphere dimension and participants’ sensory perception, emotional response, and behavioral response. This matrix facilitated the identification of significant relationships and patterns, with the intensity of these relationships represented by percentage values. The results of the query matrix were then aggregated and analyzed within a conceptual framework that links environmental aspects (stimuli) to individual behavioral responses (responses) through internal processes (mechanisms of perception, cognition, and emotion) within an individual (organism). The following section presents the findings of the query matrix analysis, which were subsequently used to synthesize themes related to the atmospheric experience.

**Table 7.** The Query Matrix of Atmosphere Dimension Elements.

Atmosphere Dimension Elements	Percentage (%)				
	1	2	3	4	5
Retail Atmosphere dimension					
Display and Decoration	35,50	44,87	30,57	33,24	50
Exterior	8,83	0	13,89	7,87	0
General Interior	16,50	8,26	22,50	22,40	7,76
Store Layout and Design	39,17	46,87	33,05	36,48	42,24
Sense					
Sight	30,67	28,31	22,17	41,64	58,54
Sound	33,36	34,55	29,77	27,42	0
Touch	21,44	21,21	16,56	24,59	25,61
Smell	7,31	1,94	13,12	6,36	0
Taste	7,23	13,99	18,37	0	15,85
Emotional Dimension					
Pleasure	69,06	53,05	69,76	57,19	22,50
Arousal	3,19	6,81	12,87	12,37	18
Dominance	27,74	40,14	17,37	30,43	59,50
Behavioral Response Theme					
Immersive and nostalgia	21,51	21,36	14,10	23,28	19,75
Personal and social interaction	27,40	22,66	16,72	16,58	16,63
Interaction with interior spaces and elements	22,72	21,86	24,25	14,77	16,40
Cultivating intention, (develop motivation to support and enhance shopping desires and goals)	22,91	26,84	16,27	16,64	17,34
Representation of identity and social affiliation	20,96	21,62	15,21	19,18	23,04

The Table 8 above summarizes the data processing results, describing the mechanism of atmospheric experience along with related aspects and their influences. A synthesis of the themes related to batik boutique atmospheres emerged from the mapping of relationships, interactions, and the effects of the intensity of retail atmosphere dimension elements, sensory perception, emotional response, and behavioral response. The themes “Cultural Intimate” and “Cozy” represent the most prominent spatial atmospheric experiences reported by participants inside the batik boutique, with an intensity of 41.37%. These are followed by the themes of “Relaxation and Connection” (24.42%) and “Holistic and Harmonic Spatial Experience” (15.93%), which focus on the general interior elements designed to create a harmonious spatial experience. Furthermore, the themes “Showing Off” and “Self-Projection” (12.23%) highlight the boutique as a space for personal expression, while the theme of “Immersive and Nostalgic” (6.06%) provides consumers with the opportunity to evoke feelings of nostalgia or comfort, such as the sensation of “feeling at home.” Based on a series of data analyses, this study focuses on the refined themes, as presented in Table 7, which outlines the sensory stimuli and spatial atmosphere within the Batik Boutique.

**Table 8.** Atmospheric Experience Theme Synthesis.

Theme	Percentage (%)
Cultural Intimate and Cozy	41,37
Relaxation and Connection	24,42
Holistic and harmonic spatial experience	15,93
Showing off and Projection of Self	12,23
Immersive and nostalgia	6,06

### 4.1 Cultural intimate and cozy

In consumer perception, the interior design arrangement and visual appeal within the boutique are critical in creating a cozy atmosphere, with less emphasis placed on external appearances. The emphasis on sight and sound suggests that these senses play a key role in fostering a comfortable and intimate environment, potentially through visual aesthetics and ambient sounds. The perception of an object through sight, touch, and sound is a significant factor in establishing a sense of intimacy and comfort with the object in question ( Schifferstein & Spence, 2008; Van Rompay & Ludden, 2015). These themes contribute to meanings that influence purchasing behavior through various emotions arising from multisensory perception and unique spatial behaviors, provided that the physical elements of the space align with the needs, motivations, memories, and values of the user. As the third participant stated, the assortment of spatial elements situated near the merchandise evoked memories of furniture design, arrangement, and decoration from her childhood home:

*“The chandeliers and wooden furniture give the room a classic feel, like something you’d find in an old-fashioned grandmother’s house. I can’t help but be drawn to these pieces, and I want to get up close and personal. The teak wood amben table with a display of batik cloths is particularly eye-catching—it’s like an instant invitation to touch and appreciate these stunning pieces.”*

The display arrangement (with products placed on the amben, a traditional wooden bench), along with the decoration, layout, and design of the space, encourages free-sitting activities and behaviors, including cross-legged and reclining positions among consumers. These elements of the retail atmosphere facilitate interaction with the products, such as displaying, stretching, observing, and touching batik. This interaction allows consumers to discuss, confer, and chat casually with friends who accompany them. As stated by Participant 1:

*“Unpacking batik on this (amben) bench is really comfortable. I can cross my legs or sit however I like, and I can truly appreciate the beauty of the batik motifs. The smell of the batik makes us feel more at home (betah) in this place and carried away by the atmosphere. If you come with friends, you can also sit and chat while looking through the batik selection, and you might even forget the time.”*

The high level of pleasure aligns with the boutique’s goal of creating a welcoming and satisfying experience, while the low level of arousal suggests a calm and relaxing atmosphere rather than an overly stimulating one. This ultimately encourages free interaction with the product and other social entities, fostering a sense of intimacy and closeness to the product. In turn, this influences consumer purchasing behavior.

### 4.2 Relaxation and connection

The experience of relaxation and connection to the spatial atmosphere in batik boutiques is primarily shaped by the flow and structure of the room. Spatial flow and sound elements contribute to a sense of relaxation and connection ( Biswas et al., 2019; Clarke et al., 2012). These atmospheric elements facilitate relaxation and enhance the ease of interaction with the batik products on display. In consumer perception, elements of display and decoration serve to reinforce the cultural and aesthetic themes of the boutique, thereby creating a strong visual appeal and artistic value associated with batik culture. As stated by Participant 4:

*“The batik displayed on the gawangan (teak wood stand/handrail) with beautiful carvings truly matches the tradition and artistic value of batik. It is very beautiful… my eyes feel satisfied and happy just looking at it.”*

The general interior dimension, while exerting a minor influence, still plays a role in establishing the initial mood as visitors enter. It fosters a sense of relaxation and comfort, though it is not a primary factor in shaping the overall atmospheric experience. Additionally, sound elements contribute to a relaxed and nostalgic ambiance, particularly through the use of background music or culturally relevant sounds. As stated by Participant 5:

*“In this room with wide door openings, I can hear the daily conversations of batik makers outside the gallery, accompanied by traditional Javanese music. It makes my heart feel more relaxed, reminding me of the atmosphere in batik craftsmen villages in Java.”*

Visual elements play a crucial role in enhancing an alluring shopping experience and reinforcing the artistic value of batik. The tactile element, which allows customers to physically interact with batik cloth, is an important factor in strengthening the emotional connection and creating a more immersive experience. Similarly, a subtle aroma enhances relaxation and comfort, though it is not the primary focus. Additionally, taste perception may be tied to cultural experiences, such as the offering of traditional drinks in the boutique to enrich the ambiance. As stated by Participant 2:

*“I love the pandan tea at this boutique. It has a very distinctive aroma and taste. I’ve heard that the recipe for this drink is a family tradition passed down from the boutique owner’s ancestors. Enjoying tea while chatting with friends feels incredibly comforting.”*

Pleasure emerges as the most dominant emotion, suggesting that the shopping experience is designed to satisfy and evoke happiness when selecting batik products. A high dominance emotion dimension indicates that shoppers feel “in control” of their shopping experience, allowing them to choose products in a relaxed and deeply connected manner. The concept of relaxation and connection can be strategically implemented by retailers to create an atmosphere that fosters both comfort and a natural connection between customers and the retail environment. Such an atmosphere enhances a welcoming shopping experience and strengthens relationships—both between customers and the products and among the customers themselves. A relaxed atmosphere fosters personal attachment to the product, which may influence purchasing decisions based on feelings of comfort and confidence. Furthermore, a relaxed setting encourages social interactions, which can positively impact purchasing behavior ( Bitner, 1992). Social connections within the boutique may further reinforce purchase decisions, as customers receive recommendations and validation from their peers, contributing to a positive and engaging shopping atmosphere.

### 4.3 Holistic and harmonic spatial experience

In the context of batik boutiques, an effective spatial arrangement naturally guides visitors while providing convenient access to the diverse batik collections, thereby creating a holistic, well-structured, and aesthetically pleasing shopping experience. The way items are displayed and decorated plays a significant role in reinforcing cultural harmony within batik boutiques. From a consumer perception perspective, the arrangement of general interior elements establishes a cohesive atmospheric foundation, fostering a sense of comfort and connection between customers and the boutique environment. As Participant 1 stated:

*“As soon as we enter this room, we feel as if we are greeted by the beautiful batik displayed on the amben, immediately making us want to touch and caress it. The height of the walls also makes us feel comfortable and familiar with the space. The openings between spaces create a sense of freedom and spaciousness, making it inviting while still feeling personal. The old terrazzo floor feels fresh (adem), and the sound of footsteps on it is calming. The beautifully carved teak wood gebyok, with its lingering woody scent, feels natural and deeply rooted in culture.”*

The exterior of the boutique serves to attract attention and provides an initial impression of the atmosphere that customers will experience upon entering. The boutique’s physical elements, including its exterior design, shape visitors’ first impressions, influencing their mood and expectations ( Bitner, 1992). From a sensory perception standpoint: (1) auditory perception responds to sound elements that enhance cultural nuances and set a relaxing and motivating mood; (2) visual perception is influenced by attractive product displays and aesthetic layouts, which contribute to a cohesive and evocative experience; and (3) tactile perception allows customers to engage directly with batik fabrics and design elements, fostering a sense of personalization and authenticity. Vision, touch, and sound perception work synergistically to create a unique and immersive connection with the boutique environment ( Bremner & Spence, 2017; Spence et al., 2014). Although its impact is indirect, taste perception can be integrated into the overall atmospheric experience, further enriching the cultural ambiance of the boutique. This concept was articulated by Participant 4, who elaborated on the role of olfactory perception, particularly the aroma of wood and tuberose. She emphasized that these scents have the power to evoke tradition, culminating in a deeper and more immersive experience. Sensory perception—encompassing sight, touch, sound, taste, and smell—creates a holistic experience that enhances customer engagement ( Spence et al., 2014). The carefully curated atmosphere plays a pivotal role in stimulating consumer interest and motivation, ultimately influencing their shopping behavior and purchasing decisions.

### 4.4 Showing off and projection of self

This study examines the formation of the theme associated with the terms “showing off” and “self-projection” within the context of batik boutiques. From a consumer perception perspective, the layout and design elements of these spaces play a pivotal role in reinforcing these themes. By strategically arranging products and optimizing displays, customers are able to move freely and perceive the shopping environment as supportive, which, in turn, enhances their confidence while shopping. The presence of culturally significant elements in displays and decorations not only enhances the visual appeal of the space but also provides customers with the opportunity to express their personal identity and values through their product choices ( Ching, 2023). As Participant 2 stated:

*“This area is quite eye-catching, with a display of beautiful batik designs on the gawangan. It’s a great spot for taking photos. The variety of batik motifs and the high quality of products displayed on the amben make us take off the fabric, look in the mirror, and then pose as if on a runway, even if it’s just rotating within this area. Usually, friends take photos and record us, then we share them on Instagram.”*

Sight is the dominant sensory modality in this experience, allowing customers to focus on batik details and cultural elements that are well-presented. This enhances their sense of pride and personal connection in selecting products. The harmonious atmosphere, created through the integration of sound elements with batik culture, fosters comfort and confidence, thereby facilitating self-expression within the boutique environment. Additionally, tactile experiences allow customers to gain a firsthand understanding of the quality of batik fabrics and interior design elements, fostering a deeper personal connection with the products. The combined perception of sight, sound, and touch supports expressions of self-projection and freedom, ultimately leading to the desire to “show off” ( Servais et al., 2022). An atmosphere that emphasizes “showing off” has been shown to encourage customers to linger longer, become more engaged with display and decoration elements, and consequently extend their visit duration. The concept of “showing off” often prompts customers to engage in photography and social media sharing of their experiences. Meanwhile, the concept of “self-projection” motivates customers to seek products that align with their personality, often leading them to spend more time exploring items that resonate with their identity. The application of these experiential concepts, namely “showing off” and “self-projection,” enables stores to create an atmosphere that not only captures attention but also fosters emotional connections and enhances customers’ sense of identity. This, in turn, can lead to an increase in purchase intentions and decisions.

### 4.5 Immersive and nostalgic

The formation of the themes of “immersive” and “nostalgic” experiences in the atmospheric space of batik boutiques is shaped by dominant display and decoration elements, such as batik arrangements, cultural ornaments, and decorative features. These elements play a crucial role in triggering nostalgic feelings and creating immersive experiences. The immersive theme, which evokes a sense of home and closeness to family, is reflected in the statement by Participant 5, who noted that product displays intentionally designed to highlight the material’s character and history can evoke addictive imagination and curiosity:

*“The way the batik is displayed on the gawangan and amben, with each piece revealing its texture and story, creates a mesmerizing effect. It’s like being pulled into its history, and I just want to know more—it feels passionate.”*

Additionally, the layout and design of the space play a significant role in fostering a sense of connection within the boutique environment. Visual perception is the most potent sensory catalyst, as it serves as a key element in evoking authenticity and nostalgia. The integration of visual and physical elements is instrumental in creating an immersive experience, thereby fostering a deep emotional connection between the consumer and the spatial environment ( Spence et al., 2014). Memorable visual elements from the past can significantly influence a person’s mood and emotions, particularly when a stimulus triggers their imagination in a given moment. For instance, nostalgia can manifest through material objects that evoke past memories and transform them into a feeling of home ( Clarke et al., 2012). As Participant 3 stated, the spatial elements surrounding the merchandise evoked memories of furniture design, arrangement, and decoration from her childhood home:

*“The decorative elements, like antique furnishings such as teak wood amben, carved teak wood gawangan, chandeliers, and traditional patterns, make me feel at home. It’s warm and familiar, almost like being surrounded by family.”*

It is essential to explore and observe elements that evoke sentiments linked to the past, as this often results in customers spending an extended duration in the store, engrossed in the process of reminiscence ( Holbrook & Schindler, 2003). The concept of the “immersive” theme has been shown to encourage customers to extend their stay in retail environments, fostering a sense of comfort and potentially facilitating interactions with staff or other patrons. A relaxed atmosphere fosters ease and engagement, prompting customers to linger longer in the store. Additionally, customers often seek to share nostalgic experiences with friends or family, either through social media or by inviting acquaintances to visit the store together. By cultivating an atmosphere that is both “immersive” and “nostalgic,” retailers can offer customers an experience that is not only sensory but also emotionally resonant. This strategy effectively captures customers’ attention and fosters loyalty, as the store successfully creates a comfortable, engaging, and memorable environment.

## 5. Discussion and contribution

### 5.1 Discussion

Retail design has gained increasing attention in academic discourse over the past two decades ( Quartier et al., 2014; Petermans & Kent, 2017), yet studies linking store atmosphere to psychological processes in consumer experiences remain limited ( Servais et al., 2022). This research highlights how batik boutiques function as more than commercial spaces, offering immersive and culturally resonant experiences. Unlike conventional retail settings that prioritize efficiency, batik boutiques encourage emotional engagement through spatial design, decorative elements, and sensory interactions. This aligns with the Stimulus-Organism-Response (SOR) paradigm ( Mehrabian & Russell, 1974), where environmental stimuli trigger emotional and behavioral responses that shape consumer experiences. The findings emphasize that consumer experiences in batik boutiques are shaped by multisensory interactions, influencing engagement, brand loyalty, and purchase intention ( Krishna, 2012; Spence et al., 2014). Visual elements, such as color, lighting, and traditional batik displays, create an aesthetic and nostalgic ambiance, reinforcing cultural authenticity. Auditory components, including background music and natural sounds, further enhance the immersive experience, influencing mood and store perception ( Biswas, 2019). Tactile engagement is another key factor, as the texture, weight, and quality of batik fabrics contribute to purchase decisions and emotional connection. Previous research suggests that physical interaction with products increases consumer attachment and purchase likelihood ( Lashkova et al., 2020). Additionally, scent and taste serve as complementary sensory layers, enriching the overall experience. The presence of traditional floral scents, wood aromas, and refreshments enhances customer satisfaction, reinforcing cultural connections and extending store visits ( Krishna et al., 2010).

Beyond sensory engagement, batik boutiques function as spaces for identity projection and social interaction. Retail design allows consumers to express personal values through product choices, reflecting a shift towards experience-driven shopping. The boutique layout and aesthetic encourage social behaviors, such as trying on products, capturing moments, and sharing experiences on digital platforms. This aligns with previous research that emphasizes retail environments as platforms for self-expression and social validation ( Bitner, 1992). The study reinforces that batik boutiques extend beyond traditional retail, offering immersive, sensory-driven, and culturally meaningful experiences. Unlike mass-market retail spaces, these boutiques emphasize heritage, storytelling, and emotional engagement, fostering brand loyalty and deep consumer attachment. Multisensory engagement enhances customer experience and brand perception, while nostalgic and immersive design elements encourage longer visits and stronger connections with the brand. Additionally, retail spaces function as platforms for self-expression and social interaction, shaping modern consumer behavior.

### 5.2 Theoretical contribution

This study contributes to the academic discourse on retail atmosphere and consumer experience by integrating multisensory engagement, cultural identity, and spatial interaction within the context of batik boutiques. While previous studies have predominantly examined store atmosphere in terms of functional and aesthetic design ( Quartier et al., 2014; Petermans & Kent, 2017), this research expands the discussion by demonstrating how culturally embedded retail spaces foster emotional connections and experiential engagement. By incorporating the Stimulus-Organism-Response (SOR) paradigm ( Mehrabian & Russell, 1974), this study reinforces the role of environmental stimuli in shaping consumer emotions and behaviors. It highlights that sensory elements such as sight, sound, touch, and scent function as psychological triggers, influencing consumer attachment, store loyalty, and purchase decisions. This research further advances the understanding of nostalgic retail design, showing how traditional decorative elements and immersive spatial arrangements enhance emotional engagement. Additionally, this study extends the experiential retail framework by positioning batik boutiques as spaces for self-expression and social interaction, aligning with contemporary theories of experiential marketing and consumer identity formation. The findings emphasize that retail spaces are not just commercial settings but also platforms for cultural preservation and personal storytelling, offering a new perspective on the intersection between retail design, cultural heritage, and consumer psychology.

### 5.3 Practical contribution

This study provides concrete guidance for retailers, particularly batik boutiques, in developing effective retail space design strategies that enhance customer experience, engagement, and brand loyalty. The findings emphasize the importance of multisensory retail design, offering practical insights for interior designers and architects to integrate lighting, color, texture, sound, and scent in creating an immersive and comfortable shopping environment. By incorporating these elements, retailers can improve customer satisfaction, encourage longer store visits, and stimulate spontaneous purchases. Additionally, this study highlights the significance of culture-centered customer experience strategies in retail design. Understanding consumer preferences and cultural identity allows retailers to create meaningful and nostalgic shopping environments. By incorporating traditional decorative elements, authentic layouts, and batik heritage narratives, boutiques can reinforce consumer appreciation for batik as a cultural legacy, strengthening their emotional connection to the brand. From a marketing and customer loyalty perspective, a strategically designed store atmosphere fosters emotional engagement and social interaction, helping retailers differentiate themselves in competitive markets. This approach is particularly relevant for businesses targeting mid-to-premium consumer segments, where shopping experiences play a vital role in consumer decision-making. Furthermore, this research serves as a framework for experience-driven retail strategies, shifting the focus from product-based retailing to emotionally enriching shopping journeys. By enhancing store ambiance, retailers can create more memorable and engaging retail spaces, contributing to long-term brand sustainability and competitiveness in the evolving marketplace.

## 6. Conclusion, limitation, and future research

This study aimed to investigates the role of multisensory design in shaping the spatial atmospheres of batik boutiques. By focusing on sensory elements—sight, sound, touch, smell, and taste—the study explores how these stimuli influence consumer experiences and emotions in boutique spaces. The findings demonstrate that batik boutiques go beyond traditional retail environments, offering immersive, culturally rich, and multisensory experiences that foster emotional connections with consumers. The integration of visual, auditory, tactile, olfactory, and gustatory elements plays a crucial role in shaping consumer perception, store attachment, and purchase intention. The study further reveals that nostalgia, identity projection, and social interaction are key factors in consumer engagement within batik boutiques. The presence of traditional decorative elements, heritage-based store layouts, and sensory-enriched atmospheres allows consumers to form meaningful psychological bonds with the space, encouraging longer visits and deeper engagement with the brand. These findings reinforce the Stimulus-Organism-Response (S-O-R) paradigm, demonstrating how environmental stimuli trigger affective and cognitive responses, ultimately influencing consumer behavior and purchase decisions.

Despite its contributions, this study has several limitations. First, the research is contextually limited to batik boutiques, which may not fully represent other segments of fashion retail. The unique cultural and artistic significance of batik may lead to different consumer responses compared to contemporary fashion stores. Lastly, consumer behavior in experiential retail is influenced by personal, social, and economic factors, which were not extensively analyzed in this study. A broader scope incorporating demographic diversity and psychological segmentation could provide a more comprehensive understanding of consumer engagement in retail spaces. Future research should explore how cultural retail spaces can adapt traditional design principles to remain relevant in an evolving global market, ensuring that emotional connections remain central to consumer engagement. Investigating how consumers form psychological bonds with retail environments is key to developing sustainable and culturally authentic shopping experiences. Lastly, exploring cross-cultural comparisons of retail atmospheres would provide valuable insights into how different cultural contexts influence consumer perceptions and behaviors, contributing to a more global understanding of experiential retail design.

## Ethical approval

This study received ethical approval from the Directorate of Research and Community Service (Direktorat Riset dan Pengabdian kepada Masyarakat/DRPM), Institut Teknologi Sepuluh Nopember, Surabaya, Indonesia, under Approval No. 1969/IT2.IV.1/B/TU.00.09/2023, dated 22 May 2023. The research strictly adhered to the ethical principles outlined in the Declaration of Helsinki ( World Medical Association, 2013), which governs ethical conduct in research involving human subjects ( https://www.wma.net/policies-post/wma-declaration-of-helsinki-ethical-principles-for-medical-research-involving-human-subjects/). Prior to participation, all individuals were thoroughly informed about the study’s purpose, procedures, potential risks, and their rights as participants. Written informed consent was obtained from each participant, confirming their voluntary participation and understanding of the study.

## Data Availability

The data supporting the findings of this study can be accessed by contacting the corresponding author ( p.setijanti@its.ac.id). Requests for data access will be considered if intended for non-commercial research purposes, with the condition that proper credit is given to the original authors. This study ensures the security and confidentiality of the data, as it involves sensitive organizational information that is classified and proprietary to the organization being studied. Since the interview participants include members of the organization’s management, there is a potential risk of reputational impact, and disclosure of the information could violate ethical research principles. To address this, the researchers have assured all participants that the data will be securely protected and used solely for research purposes. For those wishing to access the data, a formal request must be submitted to the corresponding author, accompanied by evidence of ethical approval and a detailed data access application.
