# Supplementary material for: Experiential retailing in cultural spaces: A case study of multisensory design in batik boutiques
Source: F1000Res. 2025 Jul 30;14:506. Originally published 2025 May 20. [Version 2] doi: 10.12688/f1000research.163519.2 (PMC12171720; doi:10.12688/f1000research.163519.2)
Supplement: Peer review history

# Reviewer report: Reviewer response for version 1

**DOI:** 10.5256/f1000research.179883.r386436

**Published:** 2025-06-16

**Authors:** Lusy Deasyana Rahma Devita

**Affiliations:** 1 Brawijaya University, Malang, Indonesia

This manuscript presents a timely and contextually rich investigation into the role of multisensory design in shaping consumer experiences within batik boutiques in Indonesia. The authors successfully highlight the significance of experiential retailing in culturally embedded spaces and utilize a robust qualitative methodology to explore how sensory stimuli influence emotional and behavioral responses. The application of the Stimulus-Organism-Response (S-O-R) framework is both appropriate and theoretically sound, and the findings provide meaningful insights for both academic and practical domains.

**Introduction section:**

 While the research gap has been clearly identified, the introduction would benefit from a more critical explanation of that gap. The current presentation effectively signals the underexplored nature of multisensory retailing in small-scale cultural fashion boutiques; however, the authors should go further by explicitly articulating *why* this gap matters in the broader discourse of experiential retail and what conceptual void exists in previous studies. A more critical positioning of the research problem—beyond identifying the absence—would strengthen the scholarly contribution and justify the necessity of the current study.

**Literature Review Section:**

 The literature review offers a solid theoretical foundation and discusses sensory marketing and retail atmosphere in general terms. However, it should further emphasize the *contextual nuances* of fashion boutiques operating in developing countries, especially Indonesia, where cultural identity and consumption behavior significantly differ from those in Western retail settings. This contextualization is vital to establish the relevance and originality of the case study. Moreover, integrating more *recent literature*, ideally from the past 3–5 years, would enhance the timeliness and academic rigor of the review. This includes updated empirical findings on experiential retail design in non-Western or culturally embedded retail spaces.

Is the work clearly and accurately presented and does it cite the current literature?

Yes

If applicable, is the statistical analysis and its interpretation appropriate?

Yes

Are all the source data underlying the results available to ensure full reproducibility?

Yes

Is the study design appropriate and is the work technically sound?

Yes

Are the conclusions drawn adequately supported by the results?

Yes

Are sufficient details of methods and analysis provided to allow replication by others?

Yes

Reviewer Expertise:

My research interests lie at the intersection of fashion marketing, SME strategy in the fashion industry, and behavioral science. I am particularly focused on how micro and small fashion enterprises (MSMEs) in emerging markets navigate competitive dynamics through strategic branding, multisensory retail design, and consumer behavior insights.

I confirm that I have read this submission and believe that I have an appropriate level of expertise to confirm that it is of an acceptable scientific standard.

## References

[1] : Segmenting Islamic fashion lifestyle on Indonesian woman. *Research Journal of Textile and Apparel* .2019;23(4) : 10.1108/RJTA-02-2019-0003 306-322 10.1108/RJTA-02-2019-0003

[2] : Uncovering SMEs’ knowledge management capability in innovation adoption and business performance: The moderating role of environmental turbulence. *Human Systems Management* .2024; 10.3233/HSM-230154 1-19 10.3233/HSM-230154

# Reviewer report: Reviewer response for version 1

**DOI:** 10.5256/f1000research.179883.r386427

**Published:** 2025-06-12

**Authors:** Feny Elsiana

**Affiliations:** 1 Petra Christian University, Surabaya, Indonesia

This is a good article, determining how customers' sensory experiences form patterns that assist batik boutiques' social and economic functions. The work is clearly and accurately presented.

**Methods**:  In part 3.3 of the data analysis, please highlight only the software the author used in data analysis. Adding software's reliability or the use of software in previous studies on similar topics will also deepen this part.

 In part 3.3, data analysis, please describe more of the sentence: "The results indicated a hierarchical influence of boutique elements on sensory experience." Adding a description of whether this paragraph is part of the results or the preliminary study about the hierarchical influence of boutique elements on sensory experience will enhance its clarity.

**Source data underlying the results:**

 Please provide more explanation about the case study, including the building description and the description of Areas A, B, and C.

Is the work clearly and accurately presented and does it cite the current literature?

Yes

If applicable, is the statistical analysis and its interpretation appropriate?

Yes

Are all the source data underlying the results available to ensure full reproducibility?

Partly

Is the study design appropriate and is the work technically sound?

Yes

Are the conclusions drawn adequately supported by the results?

Yes

Are sufficient details of methods and analysis provided to allow replication by others?

Yes

Reviewer Expertise:

Daylighting in architecture, sustainable architecture.

I confirm that I have read this submission and believe that I have an appropriate level of expertise to confirm that it is of an acceptable scientific standard.
